# Effectiveness of the 13-Valent Pneumococcal Conjugate Vaccine on Invasive Pneumococcal Disease in Greenland

**DOI:** 10.3390/vaccines9101123

**Published:** 2021-10-01

**Authors:** Kristiana Alexandrova Nikolova, Mikael Andersson, Hans-Christian Slotved, Anders Koch

**Affiliations:** 1 Department of Epidemiology Research, Statens Serum Institut, Artillerivej 5, DK-2300 Copenhagen S, Denmark; ASO@ssi.dk (M.A.); AKO@ssi.dk (A.K.); 2 Department of Infectious Diseases, Rigshospitalet University Hospital, Blegdamsvej 9, DK-2100 Copenhagen Ø, Denmark; 3 Department of Bacteria, Parasites & Fungi, Statens Serum Institut, Artillerivej 5, DK-2300 Copenhagen S, Denmark; HCS@ssi.dk; 4 Department of Infectious Disease Epidemiology & Prevention, Statens Serum Institut, Artillerivej 5, DK-2300 Copenhagen S, Denmark; 5 Ilisimatusarfik University of Greenland, Manutooq 1, 3905 Nuuk, Greenland; 6 Department of Internal Medicine, Queen Ingrids Hospital, 3900 Nuuk, Greenland

**Keywords:** invasive pneumococcal disease, PCV13, pneumococcal conjugate vaccine, Greenland, pneumococcal serotypes, non-vaccine serotype, vaccine serotype, streptococcal infection, herd protection, Inuit

## Abstract

The 13-valent pneumococcal conjugate vaccine (PCV13) was introduced in 2010 to the childhood vaccination program in Greenland. This study aimed to estimate the effectiveness of the PCV13 on the incidence of invasive pneumococcal disease (IPD) in children and in adults in Greenland. IPD cases from the pre-PCV13 period (January 1995–September 2010) were compared with the post-PCV13 period (September 2010–October 2020). Register data were collected from laboratory records, IPD reports, the national registry on admissions, and medical files. A total of 295 IPD cases were identified in the study period. Overall IPD incidence rate (IR) declined from the pre-PCV13 period to the post-PCV13 period (IR 23.3 to 15.3 per 100,000 person years). Overall IPD incidence among children decreased significantly, whereas overall IPD incidence among the elderly increased significantly. During the post-PCV13 period, the incidence of vaccine serotype (VT) IPD decreased in all ages, while the incidence of non-vaccine serotype (NVT) IPD increased. This increase was most substantial among elderly ≥60 years. In conclusion, the PCV13 has reduced incidence rates of IPD in Greenland. However, the increase in NVT IPD among the elderly is noteworthy, and sup-ports continued surveillance of IPD in the population of Greenland.

## 1. Introduction

Worldwide, invasive pneumococcal disease (IPD) is a major cause of morbidity and mortality with an estimated number of annual global deaths of 1.6 million people [1]. IPD occurs when the Gram-positive bacterium *Streptococcus pneumoniae* colonizes the nasopharynx via respiratory droplets and invades normally sterile sites (e.g., blood or cerebrospinal fluid), causing systemic and severe infections, such as meningitis and bacteraemia [2].

IPD incidence rates vary with time, age, geography, and population, the incidence being highest among children < 2 years, elderly > 65 years, and people with immunodeficiency disorders, underlying co-morbidities, or with high-risk behavior (e.g., smoking, alcoholism) [3,4,5]. Generally, in the Arctic regions of Alaska, Canada, and Greenland, incidence rates of IPD are higher among indigenous than non-indigenous populations [6,7,8,9]. In Greenland, the risk of IPD among Inuit is up to four times higher than in non-Inuit [6]. Moreover, the increase in incidence seems to start at an earlier age among Inuit compared to non-Inuit [7,10].

The higher incidence of IPD among indigenous populations in the Arctic regions has been found to be associated with social and environmental risk factors, including limited healthcare infrastructure, particularly in smaller, isolated settlements, domestic crowding, and comorbidity [10,11,12,13,14].

The 13-valent pneumococcal conjugate vaccine Prevnar 13® (PCV-13; Pfizer, USA) against 13 *S. pneumoniae* serotypes was the first pneumococcal conjugate vaccine to be integrated into the childhood vaccination program (CVP) in Greenland [15]. The PCV13 was introduced on the 1st of September 2010, and since then, it has been administered to children at 3 months, 5 months, and with a booster at 12 months of age, along with diphtheria, tetanus, pertussis, polio, *Haemophilus influenzae* type B, and hepatitis B vaccines [16,17].

Besides a direct protection of vaccinated children below 2 years of age, the pneumococcal conjugate vaccine has been shown to protect persons older than the targeted group for vaccination by reducing the transmission through reduced carriage of *S. pneumoniae* in the vaccinated persons, also known as ‘herd protection’ [18,19,20].

In other Arctic countries, the PCV13 vaccine has been a success, with a decline in overall IPD rates in both children and adults [21,22]. In contrast to the 7-valent pneumococcal conjugate vaccine (PCV7), which resulted in a substantial serotype replacement in the native population in Alaska [23,24], no studies among Arctic indigenous populations have found evidence of serotype replacement in IPD after the PCV13 vaccine, although the evidence among adults is unclear [21,25]. Serotype replacement refers to IPD caused by non-vaccine *S. pneumoniae* serotypes, following the introduction of a pneumococcal conjugate vaccine (PCV) [26,27,28].

There are limited data on the national epidemiology of IPD in Greenland after PCV-13 introduction, as in this period only studies on pneumococcal nasopharyngeal carriage rates have been conducted [8,14]. These studies have indicated a nasopharyngeal pneumococcal serotype shift from vaccine serotype (VT) to non-vaccine serotype (NVT) among children [8].

Thus, the impact of PCV13 introduction in Greenland on IPD rates in children and in adults is unknown.

The aim of this study was to estimate the effectiveness of the PCV13 vaccine following introduction to the CVP in Greenland in 2010 on incidence rates of IPD in children and adults.

## 2. Materials and Methods

This study is a nationwide register-based study of IPD cases retrieved from laboratory records, medical files, registered admissions to the Greenlandic National Inpatient Registry (NIR), and case reports to the National Board of Health in Greenland.

### 2.1. Setting

Greenland is the largest island in the world, but its population is small (56,367 in-habitants as of 1 July 2020) [29]. Ice covers 81% of the country [29]. All 60 settlements and 17 towns are scattered along the coastline, and transportation between towns can only be performed by ship or aircraft [29]. Most of the population is concentrated on the southwest coast which includes Nuuk, the capital, with about 18,000 inhabitants [29]. Around 89% of the population in Greenland are born in Greenland [30].

Greenland is divided into five healthcare regions with a regional hospital located in the main town of the region. There are healthcare centers in the remaining towns of the region, and healthcare stations and rural healthcare consultations in the settlements [16,17,29]. The Queen Ingrid’s Hospital (QIH) in Nuuk is the national referral hospital for all of Greenland [31]. The only microbiological laboratory in Greenland is at the QIH and receives microbiological samples for culturing from all of Greenland. After culturing, the identified *S. pneumoniae* cultures are sent to Statens Serum Institut, Copenhagen, Denmark [31], for serotyping and genotyping.

Greenland has a publicly financed healthcare system, which means that all vaccinations in the CVP are given free of charge to all with a permanent address in Greenland [16,17].

The population of Greenland have civil registration numbers (‘CPR number’), as-signed at birth and following the person throughout life. This number uniquely identifies persons in public registers, and is used to identify individuals in this study.

### 2.2. Study Population and Definitions

We included all patients who, at any point of time between 1 January 1995 and 1 October 2020, were diagnosed clinically or microbiologically, or both, with IPD. Cases were defined as patients with either an IPD diagnosis at discharge or having had *S. pneumoniae* isolated from a normally sterile site including blood, cerebrospinal fluid (CSF), synovial fluid, pericardial fluid, pleural fluid, or peritoneal fluid. Isolation of *S. pneumoniae* was positive if detected either by culture or PCR, or both, or by detection of *S. pneumoniae* antigen in urine samples (pneumococcal urinary antigen test (UAT)) from patients with a clinical diagnosis of IPD.

Patients with recurrent IPD were included if the re-occurrence of IPD was less than 30 days after the first hospitalization, or if the IPD was caused by a different serotype than the first.

We defined patients as Inuit if the persons were born in Greenland and the parents’ places of birth were unknown, or if at least one of the parents were born in Greenland. Patients were defined as non-Inuit if both parents were born outside of Greenland or, if unknown, the patient was not born in Greenland. 

### 2.3. Data Sources

We obtained data on cases with IPD from all available sources.

Cases with positive bacterial isolates of *S. pneumoniae* were identified from laboratory files from the laboratory at the QIH or from the Department of Microbiology and Infection Control at Statens Serum Institut, which serves as a microbiological reference laboratory for Greenland [31].

From the NIR in Greenland, we extracted information on all hospital admissions and discharges in Greenland for all patients who, at any point in time during the study period, were given an IPD discharge diagnosis code, according to the International Classification of Diseases (ICD) 10th and 8th revision. For patients who were not noted with a *S. pneumoniae* sample in the laboratory files, medical journals from 2002 and onwards were reviewed to confirm if microbiological demonstration of *S. pneumoniae* had been made. If not, these cases were searched for in the ‘BCC Lab’—a laboratory operator system that has held all laboratory information from all hospitals and health care stations in Greenland since 2007.

Finally, as all clinically and microbiologically verified cases of IPD are reportable by law to the Chief Medical Officer (CMO), the Greenland Board of Health, Nuuk, we scrutinized all such reporting forms.

Level of comorbidity was stratified by Charlson Comorbidty Index (CCI) score [32]. For this, means of ICD diagnosis codes, grouped by organ systems, registered on all case patients prior to their IPD discharge diagnosis were used. Three CCI groups of comorbidity were defined; low (score 0), moderate (score 1–2), and high (score > 3).

From Statistics Greenland (central statistical organization in Greenland), we obtained population denominators, and information on past and present place of residence and ethnicity, as defined by the patient’s and the parents’ places of birth, for all IPD cases.

### 2.4. Statistical Analysis

To evaluate the effectiveness of the PCV13 vaccine after its introduction in Greenland in September 2010, IPD incidence rates overall, by sex, age, ethnicity, and region of hospitalization in Greenland in the pre-PCV13 period were compared with those of the post-PCV13 period. The pre-PCV13 period spanned the interval 1 January 1995 through 31 August 2010, and the post-PCV13 period 1 September 2010 through 1 October 2020. Differences between the two study period groups were tested by chi-square test for categorical data, and by Wilcoxon test for continuous data.

To evaluate the serotype distribution between the two study periods, we grouped pneumococcal serotypes included in the PCV13 as vaccine serotypes (VT), and pneumococcal serotypes not included in the PCV13 as non-vaccine serotypes (NVT).

IPD incidence rates (IR) were calculated as the number of events per 100,000 person years (PYRS) with 95% confidence intervals (CI). To assess the impact of the PCV13, incidence rate ratios (IRR) were estimated by log-linear Poisson regression models using the glm() function in the stats package in R version 4.0.2.

A *p*-value < 5% was considered significant.

## 3. Results

### 3.1. Baseline Characteristics

A total of 295 IPD cases were identified during the study period 1 January 1995–1 October 2020; 206 cases during the pre-PCV13 period (January 1995–September 2010) and 89 cases during the post-PCV13 period (September 2010–October 2020). Fifteen patients were cases identified more than once during the entire study period, and two of these had an episode of IPD more than twice.

A total of 232 IPD case patients were identified from laboratory files from the laboratory at the QIH. A further 30 case patients were identified from the NIR, and 33 case patients from reports to the CMO. Patients who were hospitalized at the QIH in Nuuk, either directly or via transfer from a hospital or health care center outside of Nuuk, accounted for 77%, 76% and 61% of the total IPD cases identified from the above resources, respectively. 

Table 1 presents the demographic characteristics of the study groups. A total of 40% were females and 60% males. There were more females in the pre-PCV13 period than in the post-PCV13 period (43% versus 33%, *p* = 0.133).

Median age increased from 44 years (IQR 24–56 years) to 57 years (IQR 43–64 years) in the post-PCV13 period compared to the pre-PCV13 period (*p* < 0.001). A substantially larger proportion of IPD cases in the post-PCV13 period was observed among the 60–69 and ≥70 year olds compared with that in the pre-PCV13 period.

Most cases (95%) were of Inuit origin. Case patients were identified from all regions of Greenland, with the highest proportion in Nuuk (53%).

### 3.2. Clinical Characteristics

Table 2 shows the microbiological and clinical characteristics of the study population.

Meningitis and sepsis were the most common clinical diagnoses in both study groups. Meningitis occurred in more case patients during the pre-PCV13 period (36.9%) compared to the post-PCV13 period (20.2%) (*p* = 0.007), while all other clinical diagnoses occurred more often during the post-PCV13 period (*p* = 0.005).

In the post-PCV13 period, 10.1% of case patients had a high CCI score (>3), compared to the pre-PCV13 period, where 4.9% of case patients had a high CCI score (>3). This difference was not statistically significant (*p* = 0.140). Median ages were 53 and 67 years in the low and moderate CCI groups during the post-PCV13 period, compared with 42 and 56 years during the pre-PCV13 period, respectively. Median age in the high CCI group was 59 years in both the pre- and post-PCV13 periods.

### 3.3. Incidence Rates

**Figure 1:**
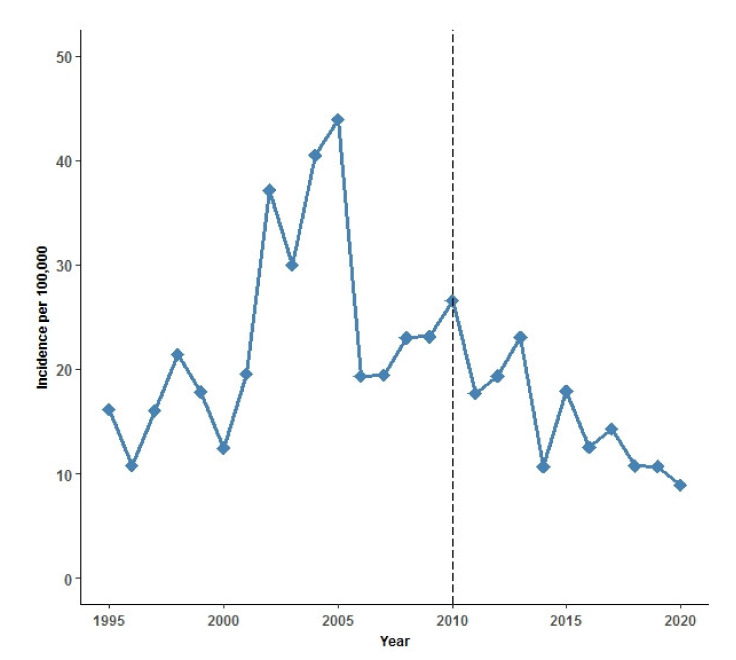
Annual incidence rate of invasive pneumococcal disease (IPD) per 100,000 person years 1995–2020 in Greenland. Black dashed line indicates introduction of the 13-valent pneumococcal conjugate vaccine (PCV13) to the childhood vaccination program (CVP) in Greenland in September 2010.

Overall, IPD incidence rates decreased from 23.3 per 100,000 PYRS in the pre-PCV13 period to 15.3 in the post-PCV13 period (*p* < 0.001) (Table 3). However, there were marked variations in incidence within the two periods. The incidence rate during the pre-PCV13 period ranged between 10 and 44, while there was a steady decline in incidence over the years in the post-PCV13 period (Figure 1).

**Figure 2:**
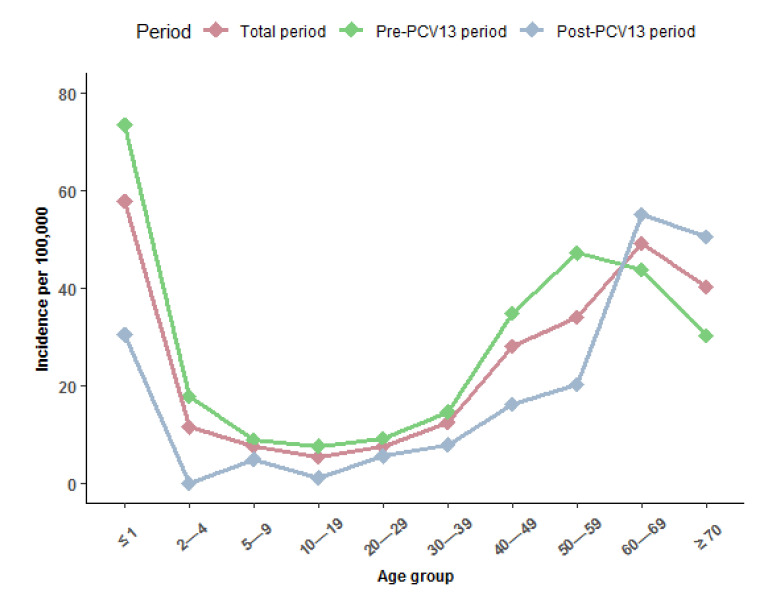
Annual incidence of invasive pneumococcal disease (IPD) per 100,000 person years by age group in Greenland 1995–2020, overall (dark pink line), pre (1995–2010) (light green line), and post (2010–2020) (gray line) introduction of the 13-valent pneumococcal conjugate vaccine (PCV13) to the childhood vaccination program in 2010 in Greenland.

The median age of IPD cases increased from the pre- to the post-PCV13 period. This was caused by the finding that IPD incidence rate decreased for all age groups, except for persons aged 60–69 years and ≥70 years (Figure 2).

The highest incidence in IPD was seen in children ≤ 1 year both during the total study period (57.8 per 100,000 PYRS) and during the pre-PCV13 period (73.5 per 100,000 PYRS). During the post-PCV13 period, the highest IPD incidence was in adults aged 60–69 (55.1 per 100,000 PYRS) (Table 3).

From the pre-PCV13 to the post-PCV13 period, the incidence of IPD among persons of Inuit ethnicity decreased, but increased in persons of non-Inuit ethnicity. Compared to other regions, the highest incidence of IPD was seen in Nuuk in both periods. The decrease in IPD incidence rates from the pre-PCV13 to the post-PCV13 period occurred in all regions (Table 3).

The incidence of meningitis in the pre-PCV13 period was 8.6 per 100,000 PYRS, which declined substantially to 3.2 per 100,000 PYRS (*p* < 0.001) in the post-PCV13 period. There was a minor decrease in the incidence of non-meningitis IPD from the pre- to the post-PCV13 period (12.6 to 12.2 per 100,000 PYRS), but this decrease was not statistically significant (*p* = 0.706) (Table 3).

### 3.4. Serotype Distribution

**Figure 3:**
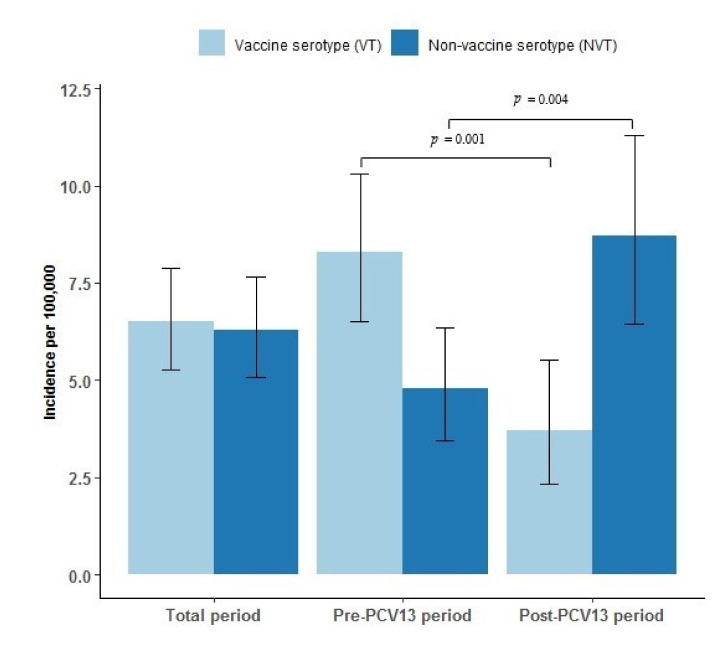
Incidence and confidence intervals of vaccine serotype (VT) and non-vaccine serotype (NVT) invasive pneumococcal disease (IPD) per 100,000 person years in Greenland 1995–2020, overall, pre (1995–2010), and post (2010–2020) introduction of the 13-valent pneumococcal conjugate vaccine (PCV13) to the childhood vaccination program in 2010 in Greenland. Confidence intervals are marked as black error bars. Differences between the pre-PCV13 and the post-PCV13 periods are marked with a horizontal black line and denote statistical significance at the *p* < 0.05 level.

Serotype determination was performed for a total of 185 case patients: for 115 case patients during the pre-PCV13 period, and for 70 case patients during the post-PCV13 period. Figure 3 shows the incidence per 100,000 PYRS of IPD by VT and NVT serotypes. The incidence of VT IPD decreased significantly in the post-PCV13 period from an incidence rate of 8.3 per 100,000 PYRS to 3.7 per 100,000 PYRS (*p* = 0.001). There were no cases at all of VT IPD after 2017 (data not shown). However, the incidence of NVT IPD increased significantly, from 4.8 per 100,000 PYRS in the pre-PCV13 period to 8.7 per 100,000 PYRS in the post-PCV13 period (*p* = 0.004).

**Figure 4:**
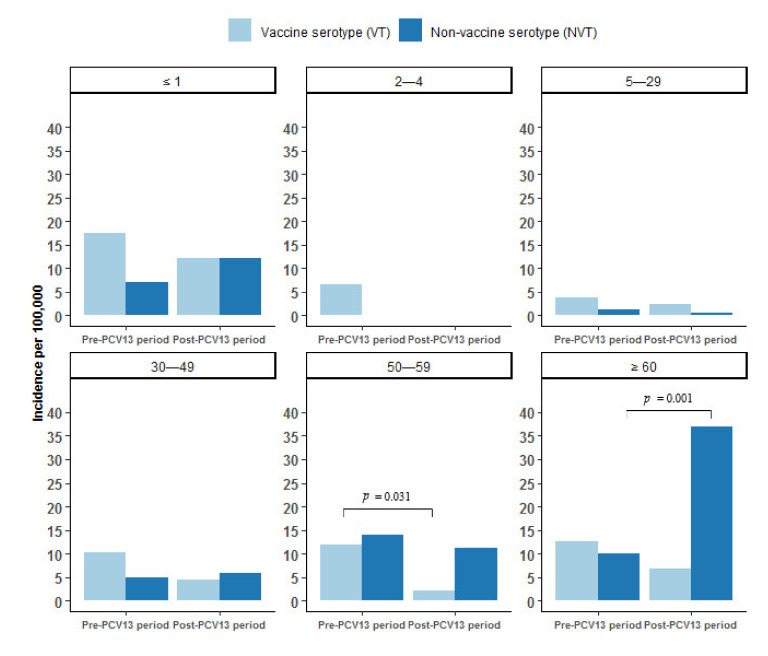
Incidence of invasive pneumococcal disease (IPD) per 100,000 person years by vaccine serotypes (VT) and non-vaccine serotypes (NVT) by age group in Greenland 1995–2020, overall, pre (1995–2010), and post (2010–2020) introduction of the 13-valent pneumococcal conjugate vaccine (PCV13) to the childhood vaccination program in 2010 in Greenland. Differences between the pre-PCV13 and the post-PCV13 periods are marked with a horizontal black line and denote statistical significance at the *p* < 0.05 level.

Figure 4 shows the incidence of IPD by VT and NVT serotypes during the pre-PCV13 and the post-PCV13 periods by age group. VT IPD decreased from the pre- to the post-PCV13 period in all age groups (statistically significant in age group 50–59 years). From the pre- to the post-PCV13 period the incidence of NVT IPD increased in age group ≤1 years, 30–49 years, and ≥60 years (statistically significant in this age group).

The most common VT serotypes during the pre-PCV13 period were serotype 1 (16%), 4 (13%), and 7F (9%). In children ≤ 4 years, the most prevalent VT serotypes during the pre-PCV13 period were 4 (10%), 6B (10%), 14 (30%), 18C (20%), and 19A (10%), which all decreased to 0% during the post-PCV13 period, except for 6B, which increased among ≤1 year olds by 36%. The most predominant VT serotypes among adults ≥60 years in the pre-PCV13 period were serotypes 3 (11%), 4 (11%), and 7F (17%), which all, except for serotype 4, decreased among adults ≥60 years during the post-PCV13 period (Supplementary Figure S1).

The most common NVT serotype during the pre-PCV13 period was 12F, which decreased markedly during the post-PCV13 period (from 13% to 3%). In children ≤1 years, the most predominant NVT serotypes during the pre-PCV13 period were 6C and 33F (14%), which both decreased during the post-PCV13 period to 0%. However, the number of NVT serotype 10A and 15C cases increased from 0% to 25% during the post-PCV13 period among ≤1 year olds. The most prevalent NVT serotypes in adults ≥60 years in the post-PCV13 period, that increased in numbers from the pre-PCV13 period, were 22F (32%), 9N (29%), 10A (14%), and 12F (7%) (Supplementary Figure S1).

## 4. Discussion

In our nationwide register-based study in Greenland, we estimated the effectiveness of the PCV13 vaccine following introduction in the CVP in 2010 in Greenland. Comparing the post-PCV13 period (2010–2020) with the pre-PCV13 period (1995–2010), we found an overall decrease in the IPD incidence rate of 30%. This reduction was most marked in children and in adults under the age of 60.

Furthermore, we found a significant reduction of 55% in overall IPD incidence caused by VT, with a complete elimination of VT IPD cases after 2017. This is consistent with findings from many countries across the world, where, following PCV13 introduction, significant reductions in VT IPD incidence have led to decreases in overall IPD incidence [4,28,33,34,35].

A very recent study found that the national coverage rate of the PCV13 in Greenland was above the WHO-recommended 90% by the first dose at 3 months of age (94.3%), but was below at the 5 (87.7%) and 12 months (83.6%) vaccinations [17]. Thus, the effects of the PCV13 could be expected to be higher with an increase in coverage rates.

The reduction in IPD incidence in children in Greenland, both overall and VT IPD, is consistent with findings in Canada, the United States, Israel, and European Countries [4,28,34,35,36,37,38,39,40]. We observed a reduction of 30% in VT IPD in children ≤ 1 year between the two periods and no VT IPD cases among 2–4 year-old children in the post-PCV13 period. It is not surprising that the reduction in IPD incidence was less among the ≤1 year olds compared with the 2–4 year olds, as the ≤1 year old children are not yet sufficiently vaccinated given the planned vaccine ages at 3, 5, and 12 months.

Even though VT IPD incidence among adults aged ≥60 years was reduced by 46% during the post-PCV13 period, the burden of VT IPD in adults persists. In many countries, moderate to substantial reductions in VT IPD among adults ≥60 years have been observed [20,28,34,37,41,42,43]. Some of these studies even showed a rapid decrease [28,34,39,41,42], even though herd protection may require from six to seven years after the implementation of the PCV13 [19].

We observed a significantly higher proportion of NVT IPD among adults ≥60 years during the post-PCV13 period, which indicates serotype replacement among the elderly in Greenland after PCV13 introduction. During the post-PCV13 period, overall IPD incidence among adults aged ≥60 years increased, which has also been observed in some European countries [37,44]. In general, the introduction of PCV13 into the CVP has been associated with an overall reduction in IPD incidence among adults, in particular those aged ≥65 years [19,45]. However, similar to our study, some studies have observed a tendency of increasing NVT IPD in adults aged ≥65 years over the PCV13 period, countering the marked declines and overall effect of the PCV13 of IPD in older adults, suggesting serotype replacement [4,20,28,36,38]. Likewise, long-term studies (up to seven years after PCV13 implementation) from many countries, including England and Wales, Canada, and France, have observed increases in overall IPD incidence rates in adults, at least four years after the implementation of the PCV13 [28,38,43]. Only some long-term studies from the United States have not observed increased IPD incidence among the elderly [35,39].

About 95% of IPD cases in our study were classified as Inuit according to place of birth. Near elimination of VT IPD in indigenous children below the age of 5 years and reductions among adults in this study are in line with other studies among Inuit populations in Alaska and Canada [21,46]. Likewise, these studies also demonstrated changes in IPD due to NVT among adults from the pre- to the post-PCV13 period, indicating serotype replacement [21,25,46,47].

Nearly all VT serotypes, except for a minor increase in serotype 6B among the ≤1 year olds, decreased in children ≤4 years from the pre- to the post-PCV13 period. Two of the most predominant VT serotypes among adults ≥60 years (serotype 3 and 7F) decreased from the pre- to the post-PCV13 period. This has also been noted in other settings where a reduction in the incidence of adult VT IPD, particularly by 7F, has been observed [27,33]. Serotype 3 has, in many countries, been observed to have a cyclical pattern [28,33,38,40]. Although figures are small, we observed the same tendency; a cyclical pattern with cases in the years 2002, 2005, 2010, 2013, and 2017.

In children ≤1 years, the most predominant NVT serotypes that had increased in incidence from the pre- to the post-PCV13 period were serotypes 10A and 15C. Serotype 10A was also observed to have increased in the post-PCV13 period among adults ≥60 years, along with serotypes 22F, 9N, and 12F. Serotypes 22F, 9N, and 12F have especially been predominant NVT serotypes among adults in a number of countries, including Canada [38,40], Israel [41], and several European countries [27,28,33,34,38,42,43,48]. Serotypes 22F, 9N, 12F, and 10A are all included in the 23-valent polysaccharide pneumococcal vaccine (PPV23) given to adults. The increase in IPD from these NVTs is a general observation in a number of countries where the burden of PPV23 serotypes increased after PCV13 implementation [28,34,36,38,41,48]. The PPV23 vaccine was, in June 2020, offered free of charge to all citizens ≥65 years in Greenland [49], but the coverage rate of this vaccine is unknown. Thus, the increase in incidence of IPD from these NVT indicates that focus on the PPV23 vaccine in adults could be of similar value in Greenland as elsewhere.

The highest incidence of IPD in our study was seen in the capital region, Nuuk. However, we do not believe this represents the true incidence of IPD in Greenland. First, all samples from hospitals and healthcare centers in Greenland must be sent to the laboratory in Nuuk. The likelihood of obtaining a laboratory-confirmed microbiological IPD diagnosis in Nuuk is therefore higher. Secondly, the transportation over large distances to the laboratory in Nuuk increases the risk of sample loss or loss of bacterial viability, which can lead to falsely negative test results. Thus, the higher incidence of IPD in Nuuk is not an entirely reliable finding, but more likely an indication of the difference in clinical practice in Greenland due to the accessibility of diagnostic possibilities [6,7,31].

Another factor of importance is that case patients during the post-PCV13 period had a higher CCI score at the same median age, which could suggest an indirect protection of the PCV13 against IPD in individuals with low CCI score [5].

The strengths of this study are its nationwide design and long study period which made it possible to capture the long-term trends in IPD epidemiology in Greenland. Moreover, we obtained a full population size, and case patients were uniquely identified on a personal identifiable level, which allowed us to obtain the exact demographic information of the study population.

There are also a number of limitations to our study. Despite the great efforts to obtain all possible IPD cases from all available sources, it was difficult to retrieve potential cases before 2002, when medical files in Greenland were computerized. In addition, it was not possible to microbiologically confirm potential IPD cases before 2007, as the national laboratory operator system ‘BCC Lab’ was not available before 2007. It is therefore possible that IPD cases during the pre-PCV13 period in our study are underreported, although we believe that the files kept by the clinical microbiological laboratory in Nuuk of IPD cases are as complete as possible.

In addition, the chance of obtaining a microbiological confirmation of an IPD diagnosis is less if the patient lives outside of Nuuk, leading to an underreporting of IPD cases outside of Nuuk. However, we do not believe that this sampling bias is largely different in the pre- compared with the post-PCV13 periods.

Only 63% of isolates were serotyped. However, isolates were cultured in Nuuk only, and such cultures had been systematically sent to Statens Serum Institut for serotyping throughout the whole study period. We therefore have no reason to believe that there was bias related to time period or region of sampling regarding isolates sent for serotyping.

Finally, while other countries implemented the PCV7 prior to the PCV13, Greenland introduced PCV13 as the first PCV in the CVP in the country. Therefore, the comparison of PCV13 effectiveness with other studies can be difficult.

## 5. Conclusions

This study found an overall reduction in IPD incidence of 30% and in VT IPD in particular (55%) following the introduction of the PCV13 in Greenland in 2010. The reductions in VT IPD incidence were observed in all age groups, and were most marked in children aged 2–4 years, where no IPD cases were observed after 2010. In contrast, we observed a remarkable increase in IPD incidence rates in the age groups ≥60 years, mainly caused by NVT IPD. This emphasizes the value of continued surveillance of IPD to maintain effective vaccination programs and possible adjustments of vaccine recommendations in the future.

## Figures and Tables

**Table 1:** Demographic characteristics of 295 patients with invasive pneumococcal disease (IPD) in Greenland 1995–2020, overall, pre (1995–2010), and post (2010–2020) introduction of the 13-valent pneumococcal conjugate vaccine (PCV13) to the childhood vaccination program in 2010 in Greenland.

	Total Period (1995–2020)	Pre-PCV13 Period (1995–2010)	Post-PCV13 Period (2010–2020)	*p*-Value ^1^
	Cases (%)	Cases (%)	Cases (%)	
No. of cases	295	206	89	
Sex ^2^				
Female	117 (39.7%)	88 (42.7%)	29 (32.6%)	0.133
Male	177 (60.0%)	117 (56.8%)	60 (67.4%)	
Age group				
Median (IQR 25%;75%)	48 (30; 59)	44 (24; 56)	57 (43; 64)	**<0.001 ^3^**
≥1	26 (8.8%)	21 (10.2%)	5 (5.6%)	0.294
2–4	8 (2.7%)	8 (3.9%)	0	0.136
5–9	9 (3.1%)	7 (3.4%)	2 (2.2%)	0.874
10–19	12 (4.1%)	11 (5.3%)	1 (1.1%)	0.173
20–29	16 (5.4%)	11 (5.3%)	5 (5.6%)	1
30–39	29 (9.8%)	23 (11.2%)	6 (6.7%)	0.338
40–49	63 (21.4%)	50 (24.3%)	13 (14.6%)	0.088
50–59	62 (21.0%)	44 (21.4%)	18 (20.2%)	0.949
60–69	49 (16.6%)	23 (11.2%)	26 (29.2%)	**<0.001**
≤70	21 (7.1%)	8 (3.9%)	13 (14.6%)	**0.002**
Ethnicity				
Inuit	279 (95.2%)	197 (96.6%)	82 (92.1%)	0.349
Non-Inuit	14 (4.8%)	7 (3.4%)	7 (7.9%)	
Region of Greenland ^4^				
Nuuk	151 (52.8%)	97 (49.0%)	54 (61.4%)	**0.044**
North	22 (7.7%)	18 (9.1%)	4 (4.5%)	0.302
South	30 (10.5%)	21 (10.6%)	9 (10.2%)	1
East	20 (7.0%)	16 (8.1%)	4 (4.5%)	0.439
West	63 (22.0%)	46 (23.2%)	17 (19.3%)	0.600

Abbreviations: PCV13: 13-valent pneumococcal conjugate vaccine. ^1^ *p*-value based on Pearson’s chi-square test for statistical difference between the pre-PCV13 and the post-PCV13 periods. Bold values denote statistical significance at the *p* < 0.05 level. ^2^ One was unknown. ^3^ *p*-value based on Wilcoxon rank sum test for statistical difference between the pre-PCV13 and the post-PCV13 periods. ^4^ North: Upernavik, Uummanaq, Qaannaaq, South: Nanortalik, Paamiut, Qaqortoq, Narsak, East: Tasiilaq, Ittoqqortoormiit, West: Aasiaat, Maniitsoq, Qasigiannguit, Ilulissat, Sisimiut, Qeqertarssuaq, Kangaatsiaq.

**Table 2:** Microbiological and clinical findings in 295 patients with invasive pneumococcal disease (IPD) in Greenland 1995–2020, overall, pre (1995–2010), and post (2010–2020) introduction of the 13-valent pneumococcal conjugate vaccine (PCV13) to the childhood vaccination program in 2010 in Greenland.

	Total Period (1995–2020)	Pre-PCV13 Period (1995–2010)	Post-PCV13 Period (2010–2020)	
	Cases (%)	Cases (%)	Cases (%)	*p*-value ^1^
*S. pneumoniae* serotypes				
Vaccine serotypes (VT)	94 (31.9%)	73 (35.4%)	21 (23.6%)	0.062
Non-vaccine serotypes (NVT)	91 (30.8%)	42 (20.4%)	49 (55.1%)	**<0.001**
Not serotyped	83 (28.1%)	68 (33.0%)	15 (16.9%)	**0.007**
Not isolated ^2^	27 (9.2%)	23 (11.2%)	4 (4.5%)	0.171
Cultured from				
Blood	187 (69.8%)	122 (66.7%)	65 (76.5%)	0.138
Cerebrospinal fluid	52 (19.4%)	41 (22.4%)	11 (12.9%)	0.097
Cerebrospinal fluid and blood	20 (7.5%)	14 (7.7%)	6 (7.1%)	1
Other ^3^	9 (3.4%)	6 (3.3%)	3 (3.5%)	1
Clinical diagnosis				
Meningitis ^4^	94 (31.9%)	76 (36.9%)	18 (20.2%)	**0.007**
Sepsis ^5^	91 (30.8%)	62 (30.1%)	29 (32.6%)	0.774
Bacteraemia ^6^	14 (4.7%)	5 (2.4%)	9 (10.1%)	0.011
Pneumonia with sepsis ^7^	37 (12.5%)	20 (9.7%)	17 (19.1%)	**0.041**
Pneumonia with bacteraemia	33 (11.2%)	22 (10.7%)	11 (12.4%)	0.827
Septic arthritis ^8^	8 (2.7%)	5 (2.4%)	3 (3.4%)	0.946
Meningitis or sepsis ^9^	18 (6.1%)	16 (7.8%)	2 (2.2%)	0.120
Charlson Comorbidity Index				
Low (0)	180 (75.3%)	127 (79.4%)	53 (67.1%)	**0.027**
Moderate (1–2)	42 (17.6%)	25 (15.6%)	17 (21.5%)	0.067
High (<3)	17 (7.1%)	8 (5.0%)	9 (11.4%)	0.140

Abbreviations: PCV13: the 13-valent pneumococcal conjugate vaccine; IPD: invasive pneumococcal disease; *S. pneumoniae*: *Streptococcous pneumoniae*; CSF: cerebrospinal fluid. ^1^ *p*-value based on Pearson’s chi-square test for statistical difference between the pre-PCV13 and the post-PCV13 periods. Bold values denote statistical significance at the *p* < 0.05 level. ^2^ Of the 27 cases without *S. pneumoniae* cultures, 2 were identified through reports to the CMO and on IPD discharge diagnoses from the NIR; 4 through reports to the CMO only; and 21 through IPD discharge diagnoses in the NIR only. ^3^ *S. pneumoniae* antigen detected in urine. One case had *S. pneumoniae* antigen detected from pericardial fluid. ^4^ Including one case with endocarditis, one with pneumonia, and one with sepsis. ^5^ Including one case with endocarditis, one with mediastinitis, and one with spondylodiscitis. ^6^ Includes one case with endocarditis, and one with perimyocarditis. ^7^ Including one case with endocarditis. ^8^ Including one case with pneumonia. ^9^ Not specified.

**Table 3:** Incidence rates and incidence rate ratios of invasive pneumococcal disease (IPD) per 100,000 person years in Greenland 1995–2020, overall, pre (1995–2010), and post (2010–2020) introduction of the 13-valent pneumococcal conjugate vaccine (PCV13) to the childhood vaccination program in 2010 in Greenland.

	Total Period (1995–2020)			Pre-PCV13 Period (1995–2010)			Post-PCV13 Period (2010–2020)				
	*n*	PYRS	IR (95% CI)	Cases	PYRS	IR (95% CI)	Cases	PYRS	IR (95% CI)	IRR (95% CI) ^1^	*p*-value ^2^
All	295	1,449,469	20.4 (18.1–22.8)	206	883,256	23.3 (20.3–26.7)	89	566,213	15.3 (12.7–19.2)	0.7 (0.5–0.9)	**0.002**
Sex ^3^											
Female	117	680,151	17.2 (14.3–20.5)	88	413,069	21.3 (17.2–26.1)	29	267,082	10.9 (7.4–15.3)	0.5 (0.3–0.8)	**0.002**
Male	177	769,319	23.01 (19.8–26.6)	117	470,187	24.9 (20.6–29.7)	60	299,131	20.1 (15.4–25.6)	0.8 (0.6–1.1)	0.175
Age group											
≤1	26	45,008	57.8 (38.3–82.9)	21	28,590	73.5 (46.3–109.5)	5	16,418	30.5 (10.9-65.50)	0.4 (0.1-1.0)	0.077
2–4	8	69,185	11.6 (5.3–21.5)	8	44,952	17.8 (8.1–33.1)	0	24,232	0		
5–9	9	117,184	7.7 (3.7–13.8)	7	77,396	9.1 (3.9–17.5)	2	39,789	5.0 (0.8-15.5)	0.6 (0.1-2.3)	0.464
10–19	12	221,989	5.4 (2.9–9.1)	11	141,832	7.8 (4.0–13.3)	1	80,156	1.2 (0.1- 5.5)	0.2 (0.0-0.8)	0.080
20–29	16	208,531	7.7 (4.5–12.1)	11	119,610	9.2 (4.8–15.8)	5	88,921	5.6 (2.0–12.1)	0.6 (0.2–1.7)	0.362
30–39	29	230,469	12.6 (8.5–17.7)	23	155,672	14.8 (9.5–21.7)	6	74,798	8.0 (3.2–16.3)	0.5 (0.2–1.3)	0.183
40–49	63	223,440	28.2 (21.8–35.7)	50	143,123	34.9 (26.1–45.5)	13	80,317	16.2 (8.9-26.6)	0.5 (0.2-0.8)	**0.014**
50–59	62	181,978	34.1 (26.3–43.3)	44	93,236	47.2 (34.6–62.5)	18	88,742	20.3 (12.3-31.2)	0.4 (0.2-0.7)	**0.003**
60–69	49	99,643	492 (36.7–63.3)	23	52,483	43.8 (28.3–62.3)	26	47,160	55.1 (36.6-79.1)	1.3 (0.7-2.2)	0.423
≥70	21	52,043	40.4 (25.5–60.2)	8	26,362	30.4 (13.9–56.5)	13	25,681	50.6 (27.8-83.3)	1.7 (0.7-4.2)	0.255
Ethnicity ^4^											
Inuit	279	1,305,472	21.4 (19.0–24.0)	197	791,603	24.9 (21.6–28.5)	82	513,870	16.00 (12.8–19.7)	0.6 (0.5–0.8)	**<0.001**
Non-Inuit	14	143,988	9.7 (5.5–15.7)	7	91,652	7.6 (3.3–14.8)	7	52,337	13.4 (5.8–25.9)	1.8 (0.6–5.1)	0.295
Region of Greenland ^5^											
Nuuk	151	401,371	37.6 (31.9–43.9)	97	225,510	43.0 (35.0–52.2)	54	175,861	30.7 (23.2–39.6)	0.7 (0.5–1.0)	0.047
North	22	162,837	13.5 (8.6–20.0)	18	103,202	17.4 (10.6–26.8)	4	59,634	6.7 (2.1–15.6)	0.4 (0.1–1.0)	0.084
South	30	241,246	12.4 (8.5–17.4)	21	156,136	13.5 (8.5–20.1)	9	85,109	10.6 (5.1–19.1)	0.8 (0.3–1.7)	0.546
East	20	89,131	22.4 (14.0–33.8)	16	55,099	29.0 (17.0–45.7)	4	34,032	11.8 (3.7–27.3)	0.4 (0.1–1.1)	0.106
West	63	554,868	11.4 (8.8–14.4)	46	343,307	13.4 (9.9–17.7)	17	211,561	8.0 (4.8–12.5)	0.6 (0.3–1.0)	0.072
Clinical Diagnosis ^6^											
Meningitis	94	1,449,469	6.5 (5.3–7.9)	76	883,256	8.6 (6.8–10.7)	18	566,213	3.2 (1.9–4.9)	0.4 (0.2–0.6)	**<0.001**
Non- meningitis	183	1,449,469	12.6 (10.9–14.5)	114	883,256	12.9 (10.7–15.4)	69	566,213	12.2 (9.5–15.3)	0.9 (0.7–1.3)	0.706

Abbreviations: PCV13: 13-valent pneumococcal conjugate vaccine, PYRS: person years, IR: incidence rate, IRR: incidence rate ratio, CI: confidence interval. ^1^ Ratios between incidence rates for the post-PCV13 period compared with the pre-PCV13 period. ^2^ *p*-value based on Pearson’s chi-square test for statistical difference between the pre-PCV13 and the post-PCV13 periods. Bold values denote statistical significance at the *p* < 0.05 level. ^3^ One was unknown. ^4^ Two were unknown. ^5^ Only 286 cases had data on region. North: Upernavik, Uummannaq, Qaanaaq, South: Nanortalik, Paamiut, Qaqortoq, Narsaq, East: Tasiilaq, Ittoqqortoormiit, West: Aasiaat, Maniitsoq, Qasigiannguit, Ilulissat, Sisimiut, Qeqertarsuaq, Kangaatsiaq. ^6^ Eighteen cases had either meningitis or sepsis. These are not included. Non-meningitis includes cases of sepsis, bacteraemia, pneumonia with sepsis, pneumonia with bacteraemia, and septic arthritis.

## Data Availability

Data on the population of Greenland are available https://www.bank.stat.gl/pxweb/en/Greenland/ (accessed on 5 November 2020). Microbiological data were acquired from the Microbiological Laboratory at the QIH, and from the Department of Microbiology and Infection Control at Statens Serum Institut, but are not publicly available. Data on course of hospitalization and comorbidity were acquired from the National Inpatient Registry in Greenland and are not publicly available.

## Supplementary Materials

The following are available online at https://www.mdpi.com/article/10.3390/vaccines9101123/s1, Supplementary Figure S1 Invasive pneumococcal disease cases by vaccine type and non-vaccine type, and age groups in Greenland 1995–2020, overall, pre (1995–2010), and post (2010–2020) introduction of the 13-valent pneumococcal conjugate vaccine in the childhood vaccination program in 2010 in Greenland.
