# Supplementary figures and images for: Effectiveness of the 13-Valent Pneumococcal Conjugate Vaccine on Invasive Pneumococcal Disease in Greenland
Source: Vaccines (Basel). 2021 Oct 1;9(10):1123. doi: 10.3390/vaccines9101123 (PMC8537731; doi:10.3390/vaccines9101123)

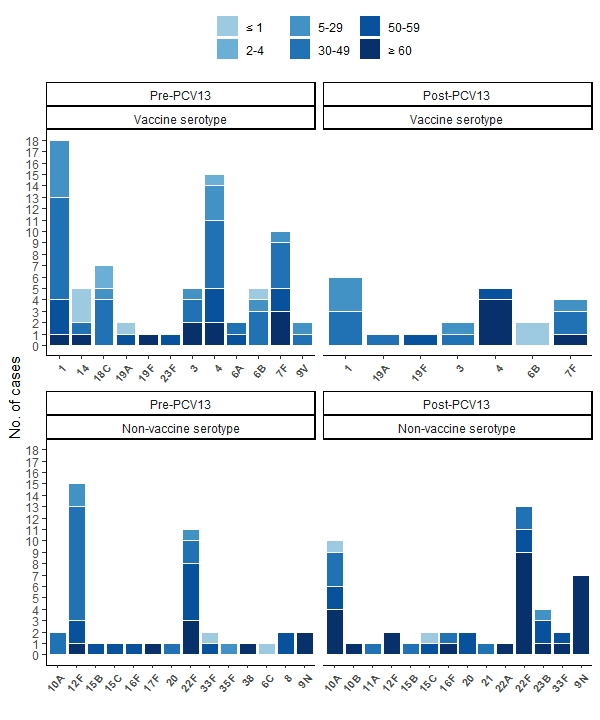

Supplement: Supplementary file 1 [file vaccines-09-01123-s001.zip › Figure S1.jpg]
